# Antenna Characteristics of Helical Coil with 2.45 GHz Semiconductor Microwave for Microwave-Enhanced Laser-Induced Breakdown Spectroscopy (MW-LIBS)

**DOI:** 10.3390/ma15082851

**Published:** 2022-04-13

**Authors:** Yuji Ikeda, Yoshihiko Hirata, Joey Kim Soriano, Ikuo Wakaida

**Affiliations:** 1 i-Lab Inc., #213 KIBC Bldg., 5-5-2 Minatojima-Minami, Chuo, Kobe 650-0047, Japan; hirata@i-lab.net (Y.H.); kim@i-lab.net (J.K.S.); 2 Remote System and Sensing Technology Division, Collaborative Laboratories for Advanced Decommissioning Science, Japan Atomic Energy Agency, 765-1 Funaishikawa, Tokai-mura, Naka-gun, Ibaraki 319-1184, Japan; wakaida.ikuo@jaea.go.jp

**Keywords:** microwave enhancements, microwave process, MW-LIBS, microwave simulation

## Abstract

A copper helical coil antenna was developed, characterized, and optimized for 2.45 GHz operations supplied by a microwave semiconductor oscillator. The application field of interest is laser-induced breakdown spectroscopy enhanced by microwave. Simulations using the Ansys HFSS demonstrate the superior localized E-field strength of the helical coil antenna, compared with other antenna-type structures. Simulation results show that E-field strength at the tip of the antenna has a logarithmic trend for increasing the coil pitch. The optimum pitch is 5 mm for a coil diameter of 6.5 mm upon consideration of the system compactness. Despite the antenna’s open-circuit end, the presence of target samples does not interfere with the E-field and H-field distribution of the antenna and the surrounding environment. Applications in microwave-enhanced laser-induced breakdown spectroscopy (MWLIBS) confirm the importance of the antenna reflector. The electric field strength was over 100 times higher than the previous capacitor-like antenna. The antenna configuration angle was then experimentally optimized for maximum enhancement effects in the spectrochemical analysis of Al_2_O_3_. The antenna angle of 60° from the laser beam propagation achieved maximum enhancement in the emission signal of Al I.

## 1. Introduction

Significant improvements in microwave semiconductor devices led to an increase in the output of pulsed power in the megawatt [1] and terawatt range [2]. These improvements are now being enjoyed by the widespread use of wireless communications powered by 5G [3] and low-cost microwave ovens. Compared to the magnetron, made of filament, tube-type device, the power value of semiconductor technology is generally low and easy to control. Previously, a pulsed drive circuit using this magnetron was attempted, which only achieved a pulse width of a few microsonds [4]. The use of semiconductor technology has made it possible to drive oscillation with nanosond width (50 ns).

We previously reported on the application of pulsed microwave to laser-induced breakdown spectroscopy (LIBS), with breakthrough enhancements in plasma size and lifetime of air and gas mixtures [5,6,7,8], and a major increase in the emission signals of solid targets, such as Al_2_O_3_ [9]. Applications of the MW-LIBS to Zr, Gd, wet Zr, and Mg were also presented [10,11,12,13,14,15]. The effect of multi-pulsed microwave in impeding the sputtering of the antenna, due to high temperatures at atmospheric pressure conditions, in contrast with a single continuous pulsed MW was also observed [9]. We call the combination of microwave and laser technology the microwave-enhanced laser-induced breakdown spectroscopy (MW-LIBS). This MW-LIBS is being applied from simple elemental analysis of laboratory scale [16,17,18,19,20,21,22,23] to detailed measurement in in-line industrial scale manufacturing [24], as well as in remote dangerous places, like inside nuclear power plants [9,10,11,12,13,14,15]. However, there are still issues regarding miniaturization, control, and cost reduction of MW-LIBS. Further improvements of MW-LIBS are needed to heed the requirements of direct applications of the system in the removal of nuclear fuel debris remaining in the Fukushima nuclear reactor following the 2011 accident [9,10,11,12,13,14,15].

Nuclear fuel debris consists of melted nuclear fuel mixed with various pieces from structures that solidified inside the reactor, estimated to be 900 tons for the three reactor units [25]. Thus, the development and storage of extraction technology has become a problem. Attempts in assisting extraction using Gamma-ray measurement, XRF, etc., have already been made [26,27]. However, durability is a problem in the context of use inside the strong radiation environment of a nuclear reactor chamber, which led to consideration of radiation-compatible optical fibers [10,11,12,13,14,15]. It is expected that the output of LIBS alone will decrease, due to deterioration of optical fibers in continuous use during the debris extraction period of several years.

Therefore, we have developed a system that efficiently transmits microwave energy to low-power transmitting LIBS [9]. We focused on the optimization of the transmitted MW through simulations and experimental optimization of the antenna characteristics. Also, MW parameter effects, such as pulse interval of the microwave and the irradiation timing of the microwave, were investigated.

Previously, a capacitor-like antenna with a chopstick structure resulted in greater than 1000 times enhancement of the Al I signal using a multi-pulse 1.9 kW peak MW oscillated at 8 ms duration [9]. In this report, we have improved the transmission efficiency of microwave energy by using a miniaturized microwave system in consideration of portability and compactness when integrated into the LIBS system [28]. In particular, the antenna shape, material, and the antenna mounting coupling was changed from using a pair of 3-mm diameter copper rods held in a 30 mm diameter coaxial connector in a chopstick-like structure and attached to an N-type connector. An inductively coupled type of antenna has been considered, using a wire diameter of 0.5 mm copper and an SMA-type connector. The shape and structure of the antenna was optimized, based on electromagnetic field simulation software ANSYS HFSS [29,30], and the actual implementation in MWLIBS through a prototyped antenna was demonstrated.

## 2. The MW-LIBS System

**Figure 1:**
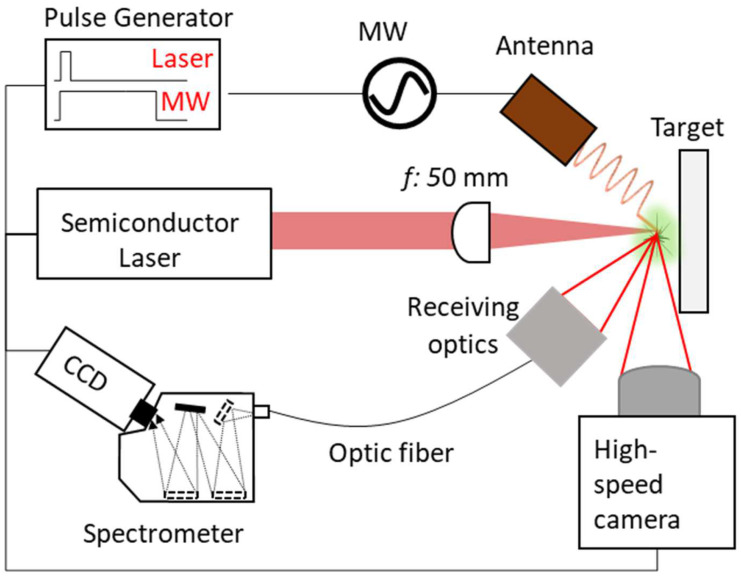
Measurement system: microwave-enhanced laser-induced breakdown spectroscopy.

The MW-LIBS is shown in Figure 1 consisting of an antenna, condensed laser, target, and receiving optics. A high-speed camera (Fastcam SA-Z, Photron, West Wycombe, UK) was also used for the visualization of the plasma. The antenna is powered by an in-house 2.45 GHz microwave semiconductor oscillator. The condensed laser is delivered by a semiconductor laser (L11038-11, Hamamatsu, Hamamatsu, Japan). The target used is standard Al_2_O_3_ (99% purity, Nilaco, Tokyo, Japan). The receiving optics consist of a 50 mm double convex lens (Thorlabs, Tokyo, Japan).

To produce the ablation plasma, the semiconductor laser irradiates 2 mJ of a 1064 nm laser beam and condenses it by a 50 mm lens into the Al_2_O_3_ sample. This is coupled with pulsed MW energy transmitted by an MW oscillator into the antenna at a repetition rate of 10 Hz. The microwave injection timing was controlled by a pulse generator (M577, Berkeley Nucleonics Corporation, San Rafael, CA, USA) with a temporal resolution of 250 ps. For the emission intensity analysis, the signal is acquired using 50 µm fiber optics, processed in an Echelle spectrometer (ME 5000, Oxford Instruments, Andor, Belfast, UK), and detected by the CCD camera (iStar ICCD-3475, Oxford Instruments, Andor, Belfast, UK).

## 3. Visualization of Plasma

In a previous report [9], it was found that when microwaves were introduced into the plasma of LIBS using a capacitor-like antenna, the emission intensity of the plasma increased. We supposed that the laser-induced plasma was sustained in space by the microwave, based on comparison of emission intensity measurements with, and without, the microwave. This time, actual time-series visualization of the microwave-enhanced laser-induced plasma was done.

**Figure 2:**
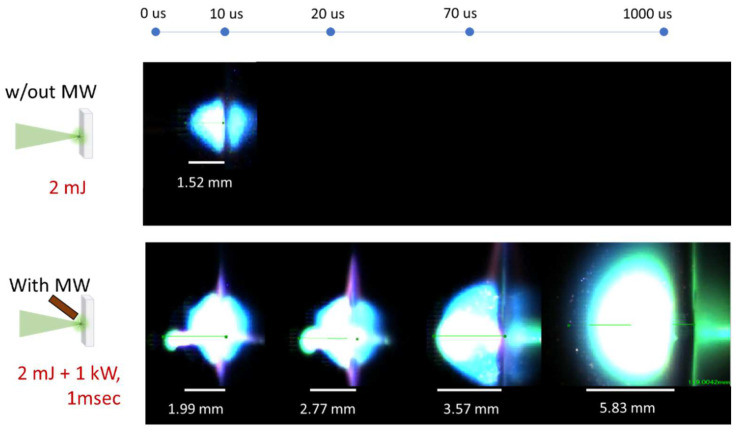
High-speed images of LIBS with, and without, the microwave.

Measurements were made at 10-microsond intervals, frame area of 640 × 280 pixels, and 100,000 frames/s. Figure 2 shows the evolution of the laser-induced plasma ablation of Al_2_O_3_ (top) and the microwave-enhanced laser-induced plasma ablation of Al_2_O_3_ (bottom). The target material was oriented vertically, and the laser was irradiated from the left side of the figure. The laser energy used was 2 mJ with a 2 ns pulse for both experiments. The microwave applied a 1.0 kW peak output for a 1.0 ms time duration. The first image frame, containing the intense propagation of the laser beam, was not shown. The sond frame after 10 μs showed clear dimensions of the plasma plume.

For laser-induced plasma ablation, the plasma plume abruptly persisted for less than 20 μs. On the other hand, microwave-enhanced laser-induced plasma showed higher plume intensity and longer sustainment period. This energy is almost 1/20 of the input energy of the previous report [9]. The laser-induced ablation plasma was sustained for several tens of μs, due to the acceleration of the electrons in the presence of MW. The microwave was emitted for 1.0 ms, and the plasma was sustained in space during that period. The plume sizes were also shown in each frame of the plasma plume. The size of the plasma irradiated with microwaves had a radius of 1.99 mm, even in the initial stage, while the size of the laser-induced plasma was 1.52 mm. Also, the size of the plasma plume sustained by the MW after 1000 μs was enlarged to 5.83 mm. The size increased to about three times that of the initial ablation size. In terms of volume, microwave-enhanced laser-induced plasma was enlarged in the cube order in comparison with laser-induced plasma. Also, in 1000 µs, the enlarged plasma was elevated from the sample surface. This suggests that the generated plasma by microwave does not contribute to the increase of ablation craters. The influence of MW on the generated craters has been investigated in detail, but will be described in a separate report [31,32].

**Figure 3:**
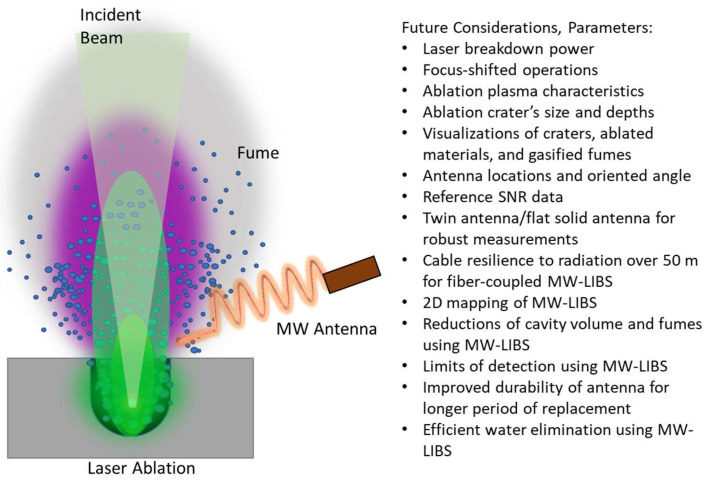
Illustration of microwave-enhanced plasma and parameters to be considered.

Based on this visualization time-series experiment, we illustrate how the laser-ablated plasma was enhanced by microwaves in Figure 3. The laser was radiated into the sample, and the microwave was emitted from the antenna which was positioned at a 60° angle with respect to the propagation of the laser. In the case of laser-induced plasma (no MW), a sharp plasma plume that filled out the space of the ablated material was formed (green). The ablation also caused the ejection of ablated particles in the form of fumes and aerosols. With the addition of microwaves, the initial plasma was magnified and enlarged (purple). We also list further parametric considerations for future analysis that have not been reported elsewhere for MW-LIBS. These parameters are summarized in Figure 3 and will be explored in future reports [32] (and maybe some readers are interested in investigating them). In this report, we are only interested in antenna optimization using EM simulations and experimental optimization of the orientation angle of the antenna.

## 4. Simulation Using HFSS

The antenna design and structure were simulated using the High-Frequency Structure Simulator (HFSS), which is an extensively used tool in the design and simulation of a wide range of antenna systems. HFSS features a high-performance full-wave electromagnetic (EM) field simulator for arbitrary 3D volumetric passive device modeling. It integrates simulation, visualization, and solid modeling in an easy-to-learn environment by employing the finite element method (FEM) and adaptive meshing. It can also be used to calculate the S parameters, electric field distribution, and magnetic field distribution.

**Figure 4:**
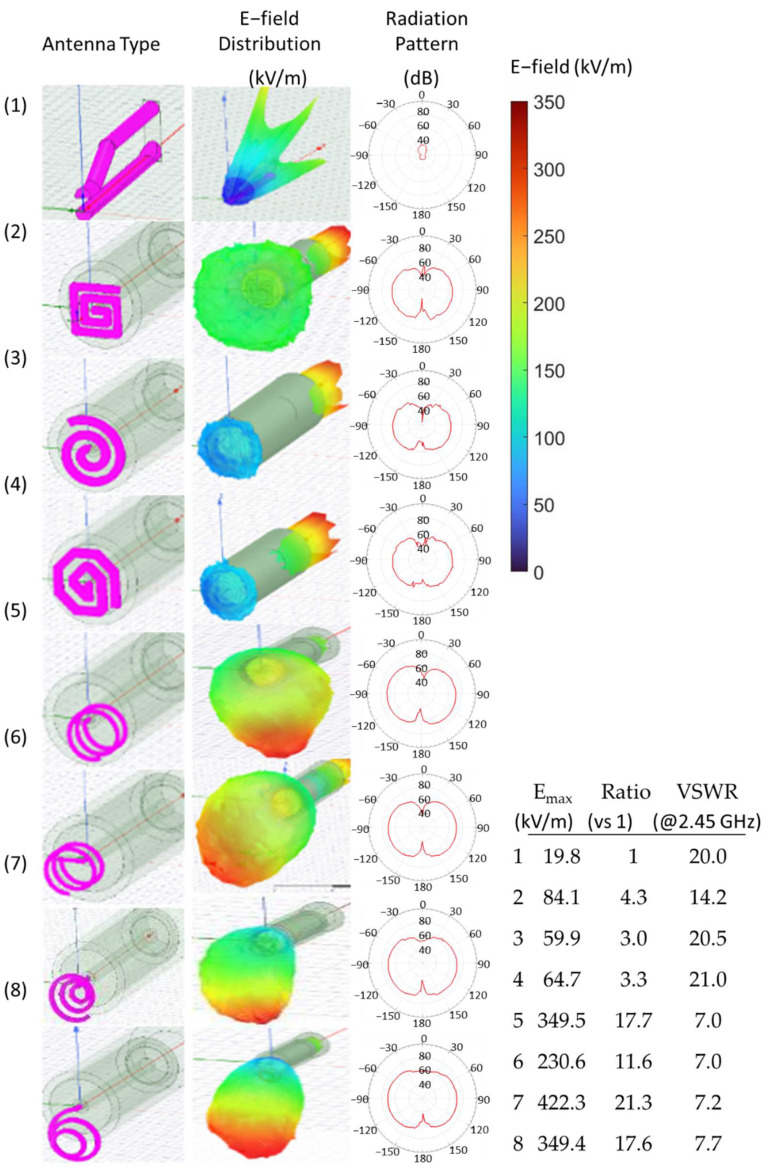
The electric field inside a spherically charged distribution and the radiation pattern of capacitor-like antenna (Antenna No. 1), square-shaped flat spiral antenna (Antenna No. 2), circular-shaped flat spiral antenna (Antenna No. 3), octagonal-shaped flat spiral antenna (Antenna No. 4), directly mounted helical coil (Antenna No. 5), bent-mounted helical coil (Antenna No. 6), conical spiral antenna with an increasing diameter (Antenna No. 7), and conical coil with decreasing diameter (Antenna No. 8). The maximum electric field strength, the ratio of the electric field versus the antenna 1, and the voltage standing wave ratio (VSWR) at 2.45 GHz for each antenna type are also shown.

Figure 4 shows the antenna simulation results using HFSS for various inductive type antennas such as flat spiral coils (Antenna Nos. 2–4), helical coils (Antenna Nos. 5–6), and conical spiral coils (Antenna Nos. 7–8). The previously used capacitor-like antenna (Antenna No. 1) is also shown as a reference. The shape and the structure of the capacitor-like antenna were previously optimized using simulations and successfully applied to MW-LIBS. Other considerations for the optimized structure include the type of material, ease of processing, performance deterioration due to deformation, mode of mounting to the connector, and ease of assembly. The copper material has been selected for all types of antennas.

In this report, we are interested in the electric field inside a spherical charge distribution and not just at the tip of the antenna. The simulation of the electric field quantitatively describes the distribution of electric field strength inside a spherical area surrounding the antenna’s tip for a microwave input of 1.0 kW peak. The simulation also shows the spatial range of EM radiation. This demonstrates the weak distribution of the electric field of the capacitor-like antenna, as compared with the inductive-type antennas. The weak E-field distribution surrounding the tip of the capacitor-like antenna indicates that the concentration of the high electric field strength in between the antenna tips is only localized in that area and rapidly dissipates at a distance. This type of localized electric field concentration is solved by the wide range emission of EM radiation by inductive-type antennas.

Three types of flat spiral antenna were designed: square-shaped (Antenna No. 2), circular-type (Antenna No. 3), and octagonal-shaped (Antenna No. 4). The square-shaped antenna showed a wider range of electric field distribution and higher maximum electric field strength at the tip of the antenna. Compared with the capacitor-like antenna, the flat panel had generally higher maximum electric field strength and a wider range of radiation, thus, it is considered to be more effective in MW-LIBS applications.

Two types of helical coil antennas were also simulated with a difference in mounting into the coaxial connector. The directly mounted helical coil antenna (Antenna No. 5) and the bent-mounted helical coil antenna (Antenna No. 6) have the same spatial range of radiation but the directly mounted helical coil antenna showed superior maximum electric field strength. Meanwhile, antennas with conical spiral shapes with increasing diameter (Antenna No. 7) and decreasing diameter (Antenna No. 8) generally showed a smaller volume of radiation, but had higher maximum electric field strengths. The radiation patterns of all the helical-type antennas were not symmetric, but the main beam was slightly skewed downwards. The skewness has something to do with the open-circuit end of the helical antennas, where the maximum localized E-field strength is located. This also explains the asymmetric distribution of EM radiation.

The radiation pattern of each antenna was also shown where all the inductive-type antennas have electric right-handed circular polarization. The directly mounted helical coil antenna was ultimately chosen for prototyping and MW-LIBS implementation, based on its superior volume of E-field distribution and lowest voltage standing wave ratio (VSWR), compared to the other types of antennas, and its highly localized E-field strength.

**Figure 5:**
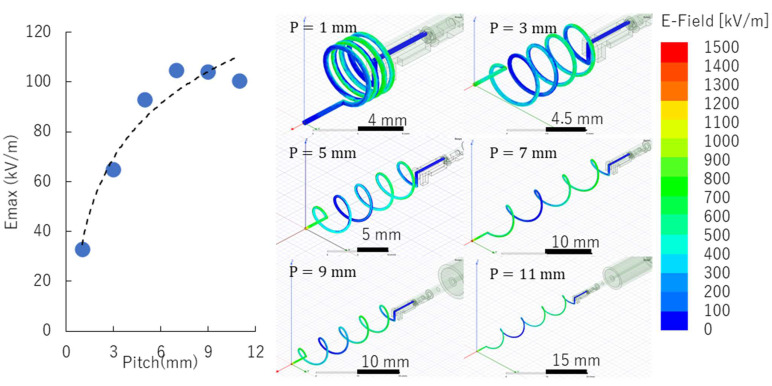
Electrical field strength and coil pitch optimization.

Further optimization of the helical coil by changing the pitch of the antenna was performed. Figure 5 shows the values of the maximum electric field at the antenna’s tip for varied coil pitches. For these simulations, copper material was used with 0.5 mm wire diameter and 5 coil turns. A pointed straight end was also considered necessary for MW-LIBS applications. A 1.0 kW, 2.45 GHz microwave power source supplied the EM radiation into the antenna. Maximum electric field strength has a logarithmic trend when the pitch is increased. A significantly high maximum electric field strength of 1200 kV/m was already achieved using the 5 mm pitch and the values slowly saturate at a 1200–1400 kV/m range with further increase in coil pitch. For compactness purposes, the 5 mm pitch was selected for the prototyped antenna. The maximum electric field strength of 1200 kV/m is almost 100 times that compared with the capacitor-like antenna used in the previous report [9].

The actual simulation results for each antenna, with varied coil pitches, were also shown. The electric field strength distribution map of all the antenna types (except for the coil pitch of 1) demonstrates maximum field strength concentrated at the tip of the antenna. This suggests that increasing the number of coil turns only increases the electric field strength at the tip of the antenna.

**Figure 6:**
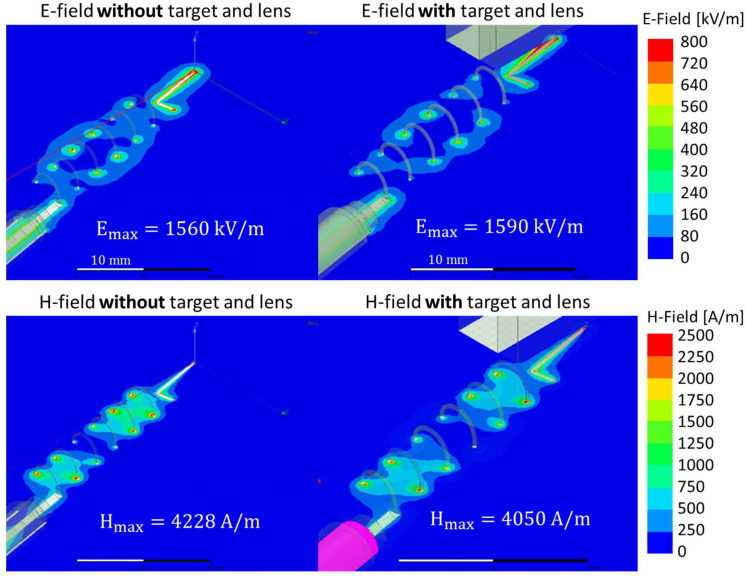
Electrical and Magnetic field strength.

Since the antenna will be implemented in the MW-LIBS, the effect of the presence of a target material and the optical lens were investigated. The target material used was Al_2_O_3_ and the lens focal length was 19.5 mm, which is one of the condensing lenses being considered for MW-LIBS. The target distance to the antenna tip was 1 mm. Figure 6 shows the electric field and magnetic field simulations for the coil pitch of 5 mm with, and without, the target and condensing lens. The results show no significant electromagnetic interference between the antenna, target material, and the lens.

## 5. Implementation of MW-LIBS

**Figure 7:**
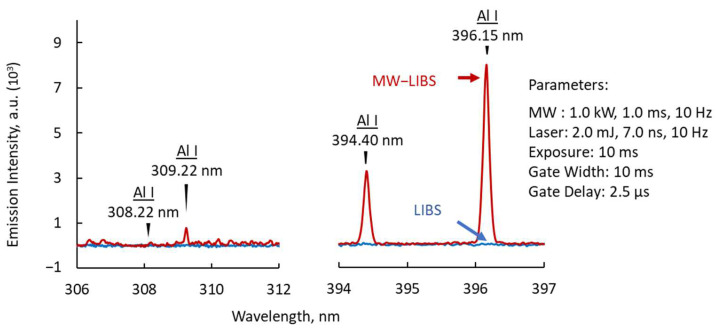
LIBS spectrum of Al with, and without, the microwave.

A prototype of the antenna was implemented in the MW-LIBS at atmospheric conditions. Figure 7 shows the emission spectrum of the laser-induced plasma with, and without, the microwave. Without the microwave, there were no visible emission peaks observed when using a gate delay of 2.5 µs. The gate delay was necessary to minimize spectral noise from the visible range. The gate delay of less than 10 µs was based on time-series visualization, which denotes a lifetime of the laser-induced plasma between 10–20 µs. With the addition of 1.0 kW, 1.0 ms pulse width microwave, four emission peaks of Al I became visible. The increase in the emission peak intensity values was nearly 1000 times higher compared to when the microwave was not turned on. The influence of background noise remained low, enhancing only the emission peaks of Al I. The process cannot be compared with photon multiplication, which affects the background spectra. The enhancement process proceeds because of the acceleration of the electrons and ions in the localized electric field generated by the microwave.

The effect of increasing the Al spectrum using the Capacitor-like Antenna was previously reported [9]. In this stion, we compare the enhancement effects of the capacitor-like antenna and the helical antenna. The optimum MW power was 1.9 kW peak using 8.0 ms MW pulse width and 30 mJ laser energy. In contrast, the helical coil antenna can only carry a limited microwave at a shorter pulse width duration. The helical coil antenna used a 1.0 kW MW peak, 1.0 ms for 0.1 mJ laser energy. When comparing the operations of the capacitor-like and helical coil antennas, almost half of the MW power and 1/8 of the pulse time duration were all that were needed to couple the microwave and the laser energy in MW-LIBS. The total microwave energy was therefore 1/16 when using the capacitor-like antenna compared with the helical coil antenna. Even though the difference may partly be due to the laser power used, an improvement in the efficiency of microwave transmission to the MW-LIBS was achieved by using the helical coil antenna where 1/16 of the previously used MW energy was successfully coupled to the laser plasma.

**Figure 8:**
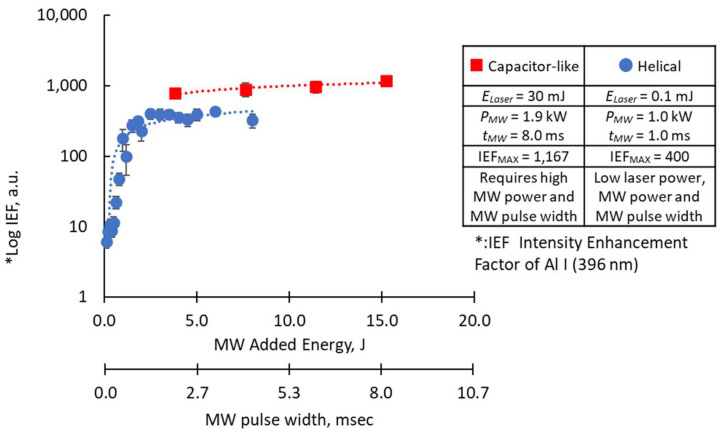
Comparison of the enhancement factors of the Al I (396 nm) emission peak when using the capacitor-like [9] and the helical antenna. The Al I signal is derived from the emission spectra of the Al_2_O_3_ breakdown plasma.

Figure 8 further compares the intensity enhancement factor of the Al *I* (396 nm) peak when using the capacitor-like antenna and the helical coil antenna. The intensity enhancement factor (*IEF*) is the ratio of the emission peak of Al *I* (396 nm) with, and without, the microwave:

(1) $$IEF=\frac{I_{PeakIntensity\;at\;396\;\mathrm{nm}}\left(with\;microwave\right)}{I_{PeakIntensity\;at\;396\;\mathrm{nm}}\left(without\;microwave\right)}$$

In the *x*-axis, microwave pulse width duration is varied, and the equivalent MW total energy is shown. The intensity enhancement factor seems to saturate microwave pulse width from 4–10 ms. Since high coupling power is needed for the capacitor-like antenna, prolonging the pulsed width may have no further impact on the intensity enhancement factor. In contrast, a helical coil antenna shows a clear logarithmic effect of the microwave pulse width and microwave total energy to the intensity enhancement factor of the Al I (396 nm) emission peak.

The helical coil antenna resulted in a maximum intensity enhancement factor of 400 times. This is lower than the 1000 times results when using the capacitor-like antenna. Even though the total energy used by both antennas was not the same, the effect of enhancing the spectrum was obtained from 0.1 mJ using the helical coil antenna. This result is vital in applications inside a nuclear reactor, where the amount of light transmitted by the optical fiber is attenuated by the effect of radiation. One of the major purposes of the integration of MW in conventional LIBS is to reinforce it. Therefore, the hundred-fold enhancement obtained in this experiment is considered sufficient for this purpose.

**Figure 9:**
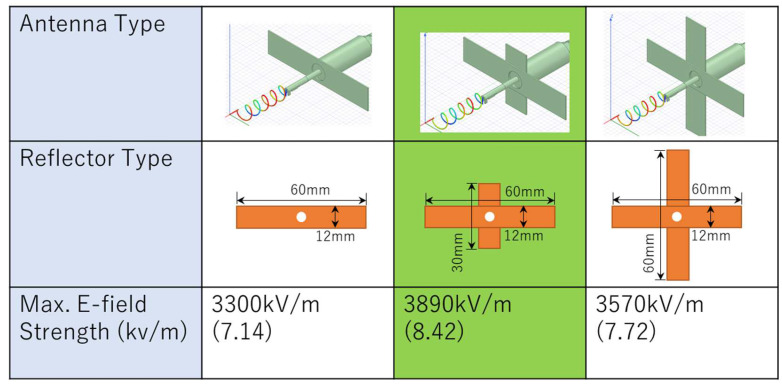
Antenna reflector design and corresponding electric field strengths.

In the design of microwave antennas, the effect of reflected waves cannot be ignored. In our system, forward and reflected microwave power is measured at the same time and sensitively controlled by a three-tuner stub. Therefore, we devised a reflector to reduce the reflector waves and lessen the use of the three-tuner stub. The reflector also offers a gain improvement of 3 dB [33]. Figure 9 shows the different designs of the antenna reflector and their corresponding maximum electric field strengths. In the HFSS simulation, the maximum electric field strength of 3500 kV/m was obtained using a cross-type plate with uneven lengths. Other shapes were also considered, such as round or rod-shaped reflectors, but the cross-type plate reflector was selected upon consideration of the ease of installation and operation.

**Figure 10:**
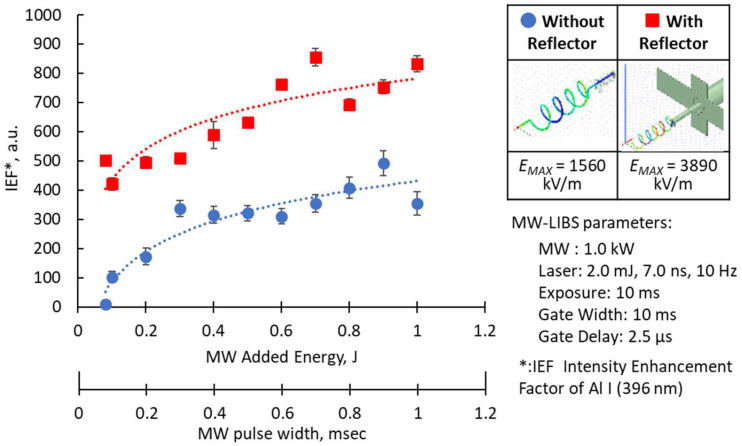
Experimental and simulated results with, and without, reflector antenna.

Figure 10 shows a comparison of the antenna with, and without, the reflector with the simulation results, and the corresponding electric field strength, indicating an increase of about three times than that when there is no reflector. The effect of enhancing the peak value of the Al spectrum in the experimental results of implementing the reflector on the antenna is shown on the left side of the figure. The intensity enhancement factor of Al I (396 nm) has logarithmic trends versus the MW added energy and pulse width, which is similar for both types of antennas. When 1.0 J of microwave was applied, the effect was almost 3 times higher when using the reflector. The increasing effect was maintained even if it decreased to 0.2 J. However, the phenomenon was unstable at 0.1 J.

From these results, the effect of the reflector was about three times higher and the effect of enhancement was maintained in the experiment regardless of the amount of microwave energy.

### Signal Enhancement Factor at a Different Microwave Input Power

**Figure 11:**
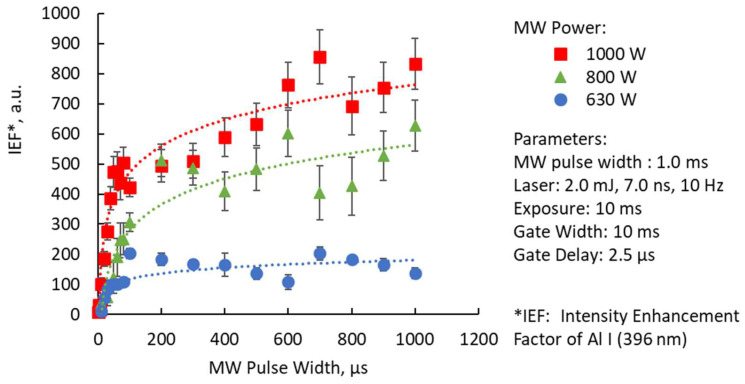
Enhancement factors for various microwave input peak powers.

The input of microwave energy varied for MW input pulse width range of 10 to 1000 μs. The MW peak value and the MW pulse width can be set from 50 to 1200 KW. This time, the peak value of the microwave input energy was changed to 1000, 800, 630 W peak. The synergistic effects of the microwave power and the MW pulse width to the intensity enhancement factor of Al I (396 nm) are shown in Figure 11. For each energy input, the logarithmic effects of MW pulse width were observed. Saturation of the enhancement factor values was observed from 100 to 1000 μs. Within this range, the results showed a consistent increase in the intensity enhancement factor with higher energy input. The microwave energy multiplied with the pulse width time duration was calculated as energy. This means that the initial energy peak from 0 to 100 μs was engaged in the enhancement of the emission peaks (increasing enhancement factor) and the remaining energy was involved in sustaining the enhanced plasma (saturated enhancement factor). In actual LIBS measurement, craters are generated and the magnitude and intensity of the initial plasma changes. Furthermore, the measurement surface is often uneven, and the magnitude, intensity, and position of the plasma generated by the laser change. The purpose of this microwave input LIBS was to obtain a certain level of enhancement effect, even with these fluctuating parameters.

**Figure 12:**
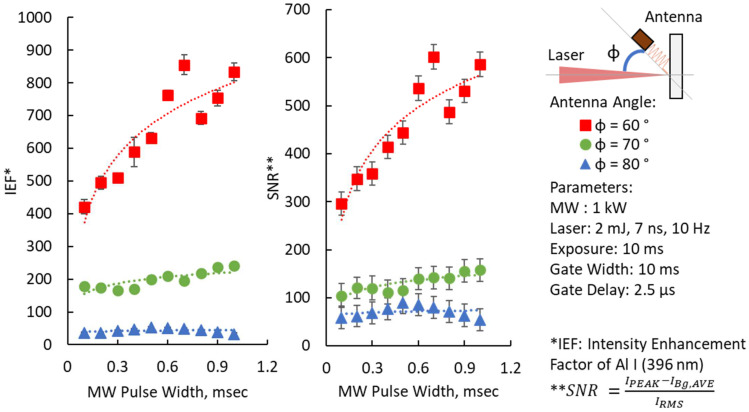
Enhancement factor and SNR for different antenna orientation angles from the laser beam propagation.

The unevenness and inclination of the measurement surface have a greater effect on the measurement result and accuracy of emission intensities; thus, we examined the antenna orientation angle effects on the intensity enhancement factor. Figure 12 shows the experimental results with antenna angle with respect to changes to 60, 70, and 80 degrees. The logarithmic effect of the MW pulse width to the intensity enhancement factor of Al I (396 nm) was again observed for each antenna angle. There seemed to be no significant enhancements when using the orientation angle of 80°. On the other hand, an almost constant enhancement effect was obtained at an angle of 70°. The optimum angle was observed at 60° with a consistently higher intensity enhancement factor and signal-to-noise (SNR) ratio compared to the other angle used.

## 6. Discussion

We prototyped a 5 mm coil pitch, 0.5 mm wire diameter, 6.5 mm coil diameter, with 5 coil turns of helical coil antenna based on the optimization results from HFSS. Based on the simulation results, the maximum localized electric field strength of the helical coil antenna is 100 times higher than that of the previously used capacitor-like antenna. The electric field strength at the tip of the antenna was observed to increase with a higher coil pitch. Other antennas considered, such as the flat spiral antenna and the conical spiral antenna, have inferior electric field strength and radiation range compared with the helical coil antenna. Despite the antenna’s open-ended circuit, the electric field strength distribution and the magnetic field strength distribution were not affected by the presence of the target sample. Further antenna optimization includes the addition of the reflector to reduce the reflected waves which also increased the electric field strength at the tip of the antenna about 3 times compared with the antenna without the reflector.

The microwave energy used in the previous report was 8.0 ms at a 1.9 kW peak, but the enhancement effect was obtained several hundred times at 0.2 ms at a 1.0 kW peak. The capacitor-like antenna was shown here as a reference because of its successful application. There are many reasons for the superior enhancement using the capacitor-like antenna, such as the higher laser energy, microwave power and pulse duration, compared with the helical coil. We did not see the need to replicate the parameters used in the previous report because the laser energy used is too large, compared with the threshold energy of 0.1 mJ. However, we tried to achieve enhancement efficiency using the helical coil antenna and reached 855 times enhancement using lower laser energy, MW power, and MW pulse width. This result is sufficient in the applications inside the nuclear reactor, where the amount of light transmitted by the optical fiber is attenuated by the effect of radiation.

The logarithmic effect of the microwave pulse duration to the intensity enhancements of the Al I (396 nm) emission peak was consistently observed for different microwave power levels, antenna types, and antenna angles. This means that the initial energy peak from 0 to 100 μs was engaged in the enhancement of the emission peaks (increasing the enhancement factor) and the remaining energy was involved in sustaining the enhanced plasma (saturated enhancement factor). The maximum intensity enhancement factor of >800 was achieved using the helical coil antenna with a reflector oriented at 60° from the laser propagation. The result was an improvement from that of the 3-loop antenna used by Khumaeni et al., which requires operation at reduced pressures [12,13,14]. In terms of the IEF and SNR values, we achieved larger values than the less than 10 times enhancement attained by Al Shuaili et al. and Wakil, using a Near-Field Applicator (NFA) with a structure of a monopole antenna [19,34]. Khumaeni et al. also achieved greater than 100 times enhancement at reduced pressure and with the aid of metastable atoms [12].

## Data Availability

Not applicable.
